# Endoscopic Hands-Off Technique versus Conventional Technique for Conversion from an Orobiliary to a Nasobiliary Tube

**DOI:** 10.1155/2016/3678647

**Published:** 2016-07-04

**Authors:** Min Jae Yang, Jae Chul Hwang, Miyeon Lee, Choong-Kyun Noh, Soon Sun Kim, Byung Moo Yoo, Jin Hong Kim

**Affiliations:** Department of Gastroenterology, Ajou University School of Medicine, Suwon 16499, Republic of Korea

## Abstract

*Background*. The aim of this study was to compare the outcomes of the endoscopic hands-off technique and the conventional technique when repositioning an endoscopic nasobiliary drainage (ENBD) tube from the mouth to the nose.* Methods*. We conducted a retrospective cohort study of all endoscopic retrograde cholangiopancreatographies (ERCPs) performed between July 2013 and May 2015 at a single tertiary referral center. A total of 1187 ERCPs were performed during the study period. Among them, 114 patients who underwent ENBD were enrolled in this study. In those patients, we used the endoscopic hands-off technique between July 2013 and May 2014 (endoscopy group) and the conventional technique between June 2014 and May 2015 (conventional group).* Results*. Technical success was achieved in 100% (58/58) of the endoscopy group and 94.6% (53/56) of the conventional group (*P* = 0.115). In the 3 cases of failed conventional technique, the endoscopic hands-off technique was then performed, and conversion of the ENBD tube was successful in all of these patients. The procedure time was significantly shorter in the endoscopy group than in the conventional group (124 s versus 149 s, *P* = 0.001).* Conclusion*. The endoscopic hands-off technique was feasible and effective for oral-nasal conversion of an ENBD tube.

## 1. Introduction

Since its introduction in 1979 [1], endoscopic nasobiliary drainage (ENBD) has been widely used for temporary biliary decompression in patients with acute suppurative cholangitis [2], postoperative bile leakage [3], or operable malignant biliary stricture [4, 5]. Placement of an ENBD tube has several advantages for the treatment of these patients after endoscopic retrograde cholangiopancreatography (ERCP), including obtaining a bile sample for bacteriological culture or cytologic analysis, monitoring bile output continuously, and flushing with water in case of clogging of the drain tube [6–8].

The insertion of an ENBD tube is an easy procedure once selective biliary cannulation is achieved. However, the rerouting of the drain tube through the nose may be difficult, especially in septic patients with confusion. In the conventional technique, a physician inserts his or her finger into the patient's oropharynx and grasps the transnasally inserted carrier tube. Then, the distal end of the tube is pulled out of the patient's mouth. During these processes, an uncooperative patient may bite the finger of the physician, leading to physical trauma or transmission of an infectious disease; further, the physician can damage the patient's oropharyngeal mucosa during the repositioning of the carrier tube [9–11]. An alternative endoscopic hands-off technique using a forward-viewing upper endoscope was initially described by Shah and Barkin [10]. This method permits grasping of a carrier tube that is placed into the oropharynx under direct vision with an endoscope and a forceps. However, no study has compared the efficacy and safety of the endoscopic hands-off technique and conventional technique. The aim of this study was to compare the outcomes between the two conversion techniques for the repositioning of an ENBD tube from the mouth to the nose.

## 2. Materials and Methods

### 2.1. Patients and Data

We conducted a retrospective cohort study of all ERCPs performed between July 2013 and May 2015 at a single tertiary referral hospital (Ajou University Hospital, Suwon, Republic of Korea). The ERCP data were collected prospectively. A total of 1187 ERCPs were performed during the study period. Among them, 114 patients who underwent ENBD were enrolled in this study. A standard dose of midazolam, propofol, and meperidine was intravenously injected for sedation during ERCP. A 7 Fr nasobiliary drainage tube (Cook Endoscopy Inc., Winston-Salem, NC, USA) was placed into the bile duct in all patients, with side-viewing endoscopes (JF-240, JF-260V, and TJF-260V; Olympus Optical Co., Ltd., Tokyo, Japan). We used the endoscopic hands-off technique from July 2013 to May 2014 (endoscopy group) and the conventional technique between June 2014 and May 2015 (conventional group) for the repositioning of an ENBD tube from the mouth to the nose. In the conventional group, the endoscopic hands-off technique was performed if technical success was not achieved with the conventional technique. This study was approved by the Institutional Review Board of Ajou University Hospital (AJIRB-MED-MDB-15-183), and informed consent was obtained from all patients before the procedure.

### 2.2. Conversion Procedures

After placement of a drainage tube inside the bile duct, the duodenoscope was withdrawn, leaving the drainage tube exiting from the mouth. A 14 Fr carrier tube (Latex suction catheter, Sewoon Medical Co., Ltd., Cheonan, Republic of Korea) was then inserted through the nose into the oropharynx.

**Figure 1:**
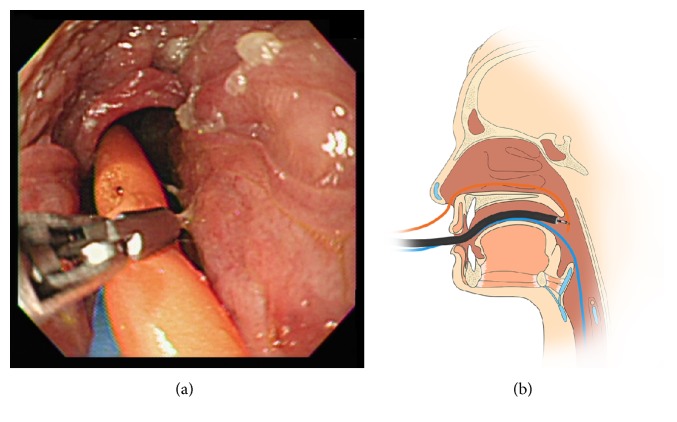
Endoscopic hands-off technique for the repositioning of an ENBD tube. (a) The distal end of a carrier tube was grasped by a rat-tooth forceps under direct endoscopic vision at the oropharynx. (b) Schematic representation of grasping the carrier tube by a rat-tooth forceps.

The endoscopic hands-off technique was performed as follows. A forward-viewing upper endoscope (GIF-XQ; Olympus Optical Co., Ltd., Tokyo, Japan), which was preloaded with a rat-tooth forceps (FG-42L-1; Olympus Corp., Tokyo, Japan), was inserted into the patient's mouth. The distal end of the carrier tube was grasped by the forceps under direct endoscopic vision at the patient's oropharynx and pulled out of the patient's mouth (Figure 1). This technique was performed by one of three endoscopists (JCH, BMY, and JHK), each of whom performs over 200 ERCP procedures per year. The end of the ENBD tube was then connected to the carrier tube. Finally, both tubes were pulled out of the patient's nose. After the procedures for oral-nasal conversion of an ENBD tube, proper positioning of the drainage tube was confirmed by fluoroscopic examination.

The conventional technique was performed as follows. One nurse who had 10 years of experience as a technical assistant in the ERCP unit, reached his finger into the oropharynx and grasped the carrier tube or changed the direction of the carrier tube from the oropharynx to the mouth while advancing the carrier tube from the patient's nose by the other free hand. The distal end of the carrier tube was pulled out of the patient's mouth. The next step was the same as the endoscopic technique.

### 2.3. Definition

Technical success was defined as the retrieval of the carrier tube from the oropharynx to the mouth and subsequent oral-to-nasal repositioning of an ENBD tube within 10 minutes. Procedure time was defined as the time elapsed from nasal intubation of the carrier tube to fluoroscopic ascertainment of proper positioning of the ENBD tube.

### 2.4. Statistical Analysis

Statistical analyses were performed using SPSS software version 18.0 for Windows (SPSS Inc., Chicago, IL, USA). Comparisons between the two groups were made using the Mann-Whitney *U* test for the continuous variables and the chi-squared or Fisher's exact test for the categorical variables. The median, minimum, and maximum were used for the continuous variables, and frequencies were used for the categorical variables. Statistical significance was set at a *P* value of < 0.05.

## 3. Results

There were no significant differences with respect to sex, age, or ENBD indication between the two groups (Table 1). Of the 58 patients in the endoscopy group, technical success was achieved in all patients and the median procedure time was 124 s (range of 50–330 s). Of the 56 patients in the conventional group, technical success was achieved in 53 (94.6%) patients and the median procedure time in the successful cases was 149 s (range of 87–410 s). There were three technical failures in the conventional group. In one patient, because the distance from the mouth to the oropharynx was longer than the length of the inserted finger, the carrier tube could not be reached at the oropharynx. In another two patients, the carrier tube repeatedly slipped on the inserted finger due to a large amount of secretion from the patient; therefore, the ENBD tube could not be pulled out of the patient's nose within 10 minutes. In all 3 patients in whom the conventional technique failed, the endoscopic hands-off technique was then performed and conversion of the ENBD tube was successful. There was no significant difference in technical success between the two groups (Table 1). The median procedure time was significantly shorter in the endoscopy group compared with the conventional group (124 s versus 149 s, *P* = 0.001). No adverse event occurred in either group during repositioning of the ENBD tube.

## 4. Discussion

In the current study, the endoscopic hands-off technique was simple, effective, and safe for conversion from an orobiliary to a nasobiliary tube. This method allowed for repositioning of the ENBD catheter in a shorter time than the conventional technique. To our knowledge, this is the first study to compare the outcomes of the endoscopic hands-off technique with the conventional technique for the repositioning of an ENBD tube from the mouth to the nose.

In the conventional technique, when pulling out the transnasally inserted carrier tube for repositioning an ENBD tube through the mouth with fingers, the patient may expectorate a large amount of secretion due to the gag reflex, and blood may ooze from an injury to the tongue or oral cavity [12]. This complication may put the patient at risk of aspiration pneumonia [12]. In addition, the physician can be exposed to the risk of biting and related trauma or to transmission of infection. ENBD is preferred by many endoscopists for temporary biliary decompression or removal of infected bile; however, repositioning the ENBD catheter through the nose may be a burden for the physician. Therefore, physicians may be unwilling to perform the procedure.

Various techniques have been reported to overcome the limitations of the conventional technique for oral-to-nasal transfer of an ENBD tube. Graepler and Gregor [13] reported a simple hands-off method for the repositioning of an ENBD tube. Instead of a forward-viewing endoscope, a laryngoscope was used to directly visualize the oropharynx. Then the carrier tube, which was introduced via the nose, was grasped with McGill forceps. Although sterilization of a laryngoscope and a McGill forceps is much simpler than reprocessing of a forward-viewing endoscope and a rat-tooth forceps, this method requires removal of the mouthpiece and special attention to not harming the uvula. Additionally, if this method fails, it is more difficult to proceed with alternative techniques because of the absence of a mouthpiece. Baron [11] described an oral-to-nasal transfer technique using a 5.4 mm ultraslim endoscope (Olympus GIF XP-160, Olympus, Center Valley, Pennsylvania, USA) in a patient in whom the carrier tube could not be passed through the nares. In this technique, the endoscope was retroflexed near the epiglottis and advanced beside the orobiliary tube out of the mouth. The end of the orobiliary tube was grasped with a pediatric retrieval basket, and the scope with the tube was withdrawn through the nasal cavity. This technique can be useful in cases in which blind insertion of a carrier tube is not possible through the nose. However, the transnasal endoscope is susceptible to biting damage, particularly in a patient without a suitable mouthpiece, because the gag reflex can be induced during passing of the retroflexed scope through the soft palate. Moreover, nasal bleeding may occur because the sharply angulated bending section of the endoscope can be compressed firmly on the nasal mucosa. In another technique using a transnasal endoscope [14], after the end of an ENBD tube was tied with a thread, the drainage tube was reinserted into the patient's oropharynx. An ultraslim endoscope was transnasally inserted into the posterior pharynx, and the thread was grasped by a biopsy forceps. Then, the ENBD tube was withdrawn from the nose. Although this method is safe for facilitating the mouth-to-nose transfer of an ENBD tube, grasping the thread with a small transnasal biopsy forceps may be technically demanding in cases involving a large amount of secretion from the patient. Watanabe et al. [15] reported the magnet-loaded catheter method for repositioning the ENBD tube from the mouth to the nose. The Nelaton tube with the daughter magnet was inserted through a nostril into the pharynx, and the suction tube with the parent magnet was inserted through the mouthpiece. The parent magnet of the suction tube attracted the daughter magnet in the Nelaton tube. Then, the Nelaton tube was pulled out of the mouth. The procedures were successful in all 20 patients without any complication; even trainees were able to perform this method safely and reliably. However, the authors of the study noted concerns about detachment of the magnet from the tube and the additional X-ray exposure that was required for the procedure. Additionally, patients with a pacemaker were excluded from this study because the magnet can affect the function of a pacemaker.

In our study, the endoscopic hands-off technique permitted direct visualization of the carrier tube, which was located in the oropharynx, as well as grasping of the carrier tube with the rat-tooth forceps, which was preloaded in the working channel of a forward-viewing endoscope. The endoscopists were able to easily perform this technique due to its simplicity. The carrier tube could be pulled out of the mouth in a short time, and the procedure time for repositioning of an ENBD tube was significantly shorter in the endoscopy group compared with the conventional group. No significant differences were observed in the technical success rates and the adverse events during repositioning of the ENBD tube between the two groups; however, the endoscopic hands-off technique was performed in 3 patients in whom the conventional technique failed, and repositioning of the ENBD tube was successful in all of these patients. The endoscopic hands-off technique can reduce the risk of trauma to physicians and patients during repositioning of the ENBD tube compared with the conventional technique. Two factors may have contributed to the similar technical success rates and adverse events between the two groups. First, the conventional technique was performed by one nurse who had 10 years of experience as a technical assistant in the ERCP unit of a tertiary referral hospital. Second, these results may reflect the relatively small number of study subjects. Reprocessing an endoscope for the endoscopic hands-off technique may be time-consuming; however, performing this procedure is not a significant burden at our center. We perform an average of 70 endoscopic examinations per day using a forward-viewing upper endoscope, and reprocessed endoscopes are always available for the next endoscopic examination. Therefore, the endoscopic hands-off technique may be applied depending on the circumstances of each center and the difficulty of each case.

Our study is limited by its retrospective design and comparison of two techniques in a nonrandomized manner. We attempted to minimize bias from these limitations by using a prospectively collected ERCP database of consecutive patients during the study period. Another limitation of the study is that it is a single-center study and that the endoscopic hands-off technique was performed by three experienced endoscopists. Large-scale, prospective, multicenter studies are needed to confirm the usefulness of this technique.

## 5. Conclusions

The endoscopic hands-off technique was feasible and effective for oral-nasal conversion of an ENBD tube. It allowed for easy repositioning of an ENBD tube without pharyngeal finger insertion and without putting either the physician or the patient at risk of trauma or complications.

## Figures and Tables

**Table 1:** Baseline characteristics and clinical outcomes of the patients.

	Endoscopic hands-off technique (*n* = 58)	Conventional technique (*n* = 56)	*P* value
Sex (male/female)	41/17	35/21	0.354
Age (years)	67 (30–91)	68 (23–96)	0.324
Indication for ENBD			0.767
Suppurative cholangitis, *n* (%)	40 (69.0)	39 (69.6)	
Incomplete stone removal, *n* (%)	11 (19.0)	6 (10.7)	
Preoperative biliary drainage, *n* (%)	6 (10.3)	10 (17.9)	
Bile leak, *n* (%)	1 (1.7)	1 (1.8)	
Technical success, *n* (%)	58 (100.0)	53 (94.6)	0.115
Procedure time (sec)	124 (50–330)	149 (87–410)	0.001

The data are presented as the numbers of patients or as the median (range).

ENBD: endoscopic nasobiliary drainage.
